# Enhanced perioperative care in emergency general surgery: the WSES position paper

**DOI:** 10.1186/s13017-023-00519-2

**Published:** 2023-10-06

**Authors:** Marco Ceresoli, Marco Braga, Nicola Zanini, Fikri M. Abu-Zidan, Dario Parini, Thomas Langer, Massimo Sartelli, Dimitrios Damaskos, Walter L. Biffl, Francesco Amico, Luca Ansaloni, Zsolt J. Balogh, Luigi Bonavina, Ian Civil, Enrico Cicuttin, Mircea Chirica, Yunfeng Cui, Belinda De Simone, Isidoro Di Carlo, Andreas Fette, Giuseppe Foti, Michele Fogliata, Gustavo P. Fraga, Paola Fugazzola, Joseph M. Galante, Solomon Gurmu Beka, Andreas Hecker, Johannes Jeekel, Andrew W. Kirkpatrick, Kaoru Koike, Ari Leppäniemi, Ingo Marzi, Ernest E. Moore, Edoardo Picetti, Emmanouil Pikoulis, Michele Pisano, Mauro Podda, Boris E. Sakakushev, Vishal G. Shelat, Edward Tan, Giovanni D. Tebala, George Velmahos, Dieter G. Weber, Vanni Agnoletti, Yoram Kluger, Gianluca Baiocchi, Fausto Catena, Federico Coccolini

**Affiliations:** 1 School of Medicine and Surgery, Milano-Bicocca University, Monza, Italy; 2 General and Emergency Surgery Department, Fondazione IRCCS San Gerardo dei Tintori, Via Pergolesi 33, 20900 Monza, Italy; 3 General Surgery Department, Bufalini Hospital, Cesena, Italy; 4 The Research Office, College of Medicine and Health Sciences, United Arab Emirates University, Al Ain, UAE; 5 General Surgery Department - Santa Maria Della Misericordia Hospital, Rovigo, Italy; 6 Department of Anesthesia and Critical Care, ASST Grande Ospedale Metropolitano Niguarda, Milan, Italy; 7 General Surgery, Macerata Hospital, Macerata, Italy; 8 Department of General Surgery, Royal Infirmary of Edinburgh, Edinburgh, UK; 9 Scripps Memorial Hospital La Jolla, La Jolla, CA USA; 10 John Hunter Hospital Trauma Service and School of Medicine and Public Health, The University of Newcastle, Newcastle, AU Australia; 11 General Surgery, Fondazione IRCCS San Matteo, Pavia, Italy; 12 Department of Traumatology, John Hunter Hospital and University of Newcastle, Hunter Medical Research Institute, Newcastle, NSW Australia; 13 Division of General and Foregut Surgery, Department of Biomedical Sciences for Health, IRCCS Policlinico San Donato, University of Milan, Milan, Italy; 14 University of Auckland, Auckland, New Zealand; 15 Department of Digestive Surgery, CHU Grenoble Alpes, Grenoble, France; 16 Department of Surgery, Tianjin Nankai Hospital, Nankai Clinical School of Medicine, Tianjin Medical University, Tianjin, China; 17 Unit of Emergency and Trauma Surgery, Villeneuve St Georges Academic Hospital, Villeneuve St Georges, France; 18 Department of Surgical Sciences and Advanced Technologies, General Surgery Cannizzaro Hospital, University of Catania, Catania, Italy; 19 PS_SS Weissach im Tal, Weissach im Tal, Germany; 20 Department of Critical Care and Anesthesia, Fondazione IRCCS San Gerardo Dei Tintori, Monza, Italy; 21 Division of Trauma Surgery, School of Medical Sciences (FCM), University of Campinas (Unicamp), Campinas, Brazil; 22 University of California, Davis, Sacramento, CA USA; 23 University of Otago, Dunebin, New Zealand; 24 Department of General and Thoracic Surgery, University Hospital of Giessen, Gießen, Germany; 25 Erasmus MC University, Rotterdam, The Netherlands; 26 General, Acute Care, Abdominal Wall Reconstruction, and Trauma Surgery, Foothills Medical Centre, Calgary, AB Canada; 27 Department of Primary Care and Emergency Medicine, Kyoto University Graduate School of Medicine, Kyoto, Japan; 28 Helsinki University Hospital and University of Helsinki, Helsinki, Finland; 29 Andrei Litvin, CEO AI Medica Hospital Center, Kaliningrad, Russia; 30 Department of Trauma, Hand, and Reconstructive Surgery, Goethe University, Frankfurt University Hospital, Frankfurt am Main, Germany; 31 Director of Surgery Research, Ernest E. Moore Shock Trauma Center, Distinguished Professor of Surgery, University of Colorado, Denver, CO USA; 32 Department of Anesthesia and Intensive Care, Parma University Hospital, Parma, Italy; 33 Third Department of Surgery, Attikon University Hospital, Athene, Greece; 34 General Surgery, ASST Papa Giovanni XXIII, Bergamo, Italy; 35 Department of Surgical Science, University of Cagliari, Cagliari, Italy; 36 RIMU Plovdiv/UMHAT St George Plovdiv, Plovdiv, Bulgaria; 37 Department of General Surgery, Tan Tock Seng Hospital, Singapore, Singapore; 38 Department of Surgery, Brody School of Medicine, East Carolina University, Greenville, NC USA; 39 Former Chair Department of Emergency Medicine, HEMS Physician, Radboud University Medical Center, Nijmegen, The Netherlands; 40 Digestive and Emergency Surgery Department, Azienda Ospedaliera S.Maria, Terni, Italy; 41 Harvard Medical School - Massachusetts General Hospital, Boston, USA; 42 Department of General Surgery, Royal Perth Hospital, Head of Service and Director of Trauma, Royal Perth Hospital, The University of Western Australia, Perth, Australia; 43 Anesthesia and Critical Care Department, Bufalini Hospital, Cesena, Italy; 44 Department of General Surgery, The Rambam Academic Hospital, Haifa, Israel; 45 General Surgery, University of Brescia, ASST Cremona, Cremona, Italy; 46 Emergency Surgery, University of Pisa, Pisa, Italy

## Abstract

Enhanced perioperative care protocols become the standard of care in elective surgery with a significant improvement in patients’ outcome. The key element of the enhanced perioperative care protocol is the multimodal and interdisciplinary approach targeted to the patient, focused on a holistic approach to reduce surgical stress and improve perioperative recovery. Enhanced perioperative care in emergency general surgery is still a debated topic with little evidence available. The present position paper illustrates the existing evidence about perioperative care in emergency surgery patients with a focus on each perioperative intervention in the preoperative, intraoperative and postoperative phase. For each item was proposed and approved a statement by the WSES collaborative group.

## Introduction

Enhanced recovery after surgery (ERAS®) protocol refers to a standardized multimodal approach based on the application of structured protocols in perioperative patients' management. The main goal of these interventions is patient management optimization during the perioperative period under all aspects of perioperative care, not only about the surgical technique, by reducing surgical stress, minimizing the physiological response to surgery, and improving postoperative recovery. The key element of the ERAS protocol is the multimodal and interdisciplinary approach targeted to the patient, focused on a holistic approach [1].

**Fig. 1:**
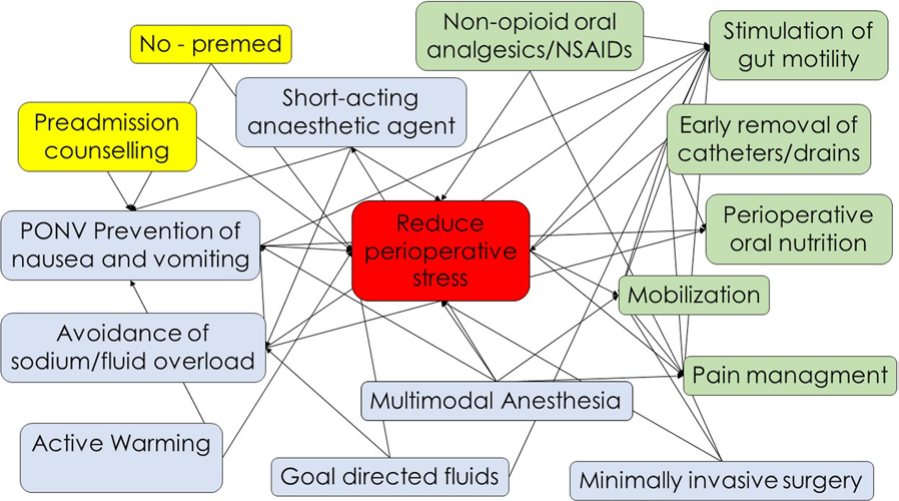
Enhanced perioperative care items and interventions

Perioperative care protocols are structured as a bundle of interventions to be applied during the preoperative, intraoperative and postoperative periods. Each intervention is linked to the others and shares the common goal of reducing the burden of perioperative patient stress (Fig. 1). For this reason, it is very difficult to evaluate the efficacy of a single item without considering the effect of all the others, applied as a bundle. Designing studies to evaluate and demonstrate the effect of every single intervention is therefore a major challenge, markedly limiting the available scientific evidence.

The effectiveness and safety of ERAS protocols in elective surgery are now widely established. Several meta-analyses comparing standard care and fast-track approach show that ERAS protocols in elective surgery lead to a reduction in length of stay and in the rate of postoperative non-surgical complications [2–5]. Most of the available studies focused on the postoperative phase, considering the main “surgical” items as study outcomes, with relatively small attention being devoted to preoperative and intraoperative interventions. However, main postoperative items such as oral feeding, urinary drain removal and mobilization should also be considered as compliance indicators rather than only interventions to be implemented [6]. From a methodological standpoint, there is a clear difference between adherence and compliance to an enhanced recovery protocol. *Adherence* should identify the percentage of items applied throughout the perioperative care process, while *postoperative compliance* also reflects how the patient follows the enhanced recovery process. For example, patients’ compliance to a postoperative pathway including early oral feeding and mobilization can be obtained easier if there is good adherence to a preoperative and an intraoperative enhanced pathway (and not only for a medical decision).

Based on the beneficial effect of enhanced perioperative care protocols in elective surgery, the implementation of structured protocols for emergency general surgery patients has also been advocated after the promising results of some studies [7, 8]. However, enhanced perioperative care in emergency general surgery remains a “grey area” with little evidence available and great debate.

**Fig. 2:**
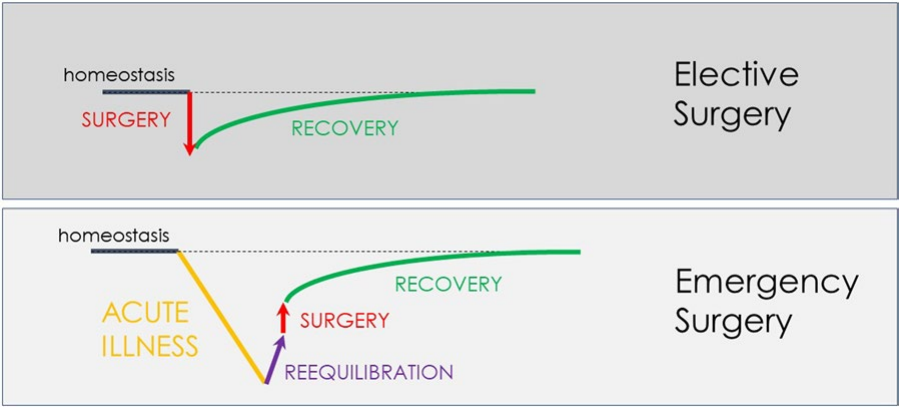
Perioperative diagram of patient’s homeostasis in elective and emergency general surgery

Patients undergoing elective surgery should be normothermic, euvolemic, clean, and “healthy”, and surgery per se represents their main stressful factor. Emergency surgery represents a more complex scenario where surgery is at the same time a stressful factor but also the key-intervention to solve the pre-existing physiologic imbalance secondary to the acute underlying disease (Fig. 2).

The diagram (Fig. 2) shows the impact of the pre-existing acute disorder causing a marked decline of the physiological reserve. The importance of the preoperative phase (re-equilibration) seems intuitive. Despite emergency surgery by definition does not allow schedulable interventions, some preoperative optimization is still possible in the emergency setting, though with much reduced time. The time available between patient presentation and surgery should be optimized to improve the patient’s physiological status to promote post-surgical recovery. In this complex scenario, also the timing of surgery should be carefully evaluated. One of the most intriguing and difficult challenges is to identify the right balance between hastening surgery to directly "face" the acute disease and delaying surgery in trying to improve the patient's condition.

## Evidence supporting enhanced perioperative care in emergency general surgery.

Currently, available studies about enhanced perioperative care in emergency general surgery are few, sparse and very heterogeneous. In addition, the perioperative care protocol derived from the elective ERAS protocol but with several and substantial differences [9–28]. Tables 1 and 2 show in detail the protocols adopted in the evaluated studies. A single perioperative care protocol cannot be identified through the existing literature, and each study applied different interventions.

**Table 1:** Intraoperative and postoperative protocols description among studies on emergency general surgery

	Study design	Patients	Number of patients	Preoperative		Intraoperative					
				Counselling	preoperative resuscitation	PONV prophylaxis	Avoiding hypothermia	Fluid management	Analgesia	Monitoring of neuromuscolare blockade	Anesthesia depth monitoring
Burchart 2021	Prospective observational study	Major abdominal surgery	227	n/d	n/d	Dexamethasone 16 mg + ondansetron 4 mg	Active warming (core temperature > 36 °C)	MAP guided fluid therapy (MAP > 65 mmHg)	TIVA + epidural or TAP block	TOF	n/d
Ceresoli 2023	Prospective observational study	Gastrointestinal surgery	589	n/d	N/A	Desamethasone 4 mg	Active warming with warm air	MAP guided fluid therapy (MAP > 65 mmHg)	Epidural or TAP block	TOF	Described
Chndan 2021	RCT	Perforated peptic ulcer	42/43	n/d	n/d	n/d	n/d	n/d	n/d	n/d	n/d
Gonenc 2013	RCT	Perforated peptic ulcer	26/21	n/d	n/d	Indicated but n/d	n/d	n/d	n/d	n/d	n/d
Lohsiriwat 2014	Observational case–control study	Obtructive colorectal	20/40	Preoperative counselling	n/d	Indicated but n/d	Active warming with warm air	n/d	Trocar site infiltration with bupiivacaine	n/d	n/d
Lohsiriwat 2019	Retrospective observational study	Colorectal	46/14	Preoperative counselling	n/d	Indicated but n/d	Active warming with warm air	Near zero balance	Balanced opioid sparing	n/d	n/d
Masood 2021	RCT	Perforated duodenal ulcer	16/18	n/d	n/d	n/d	n/d	n/d	n/d	n/d	n/d
Mohsina 2018	RCT	Perforated peptic ulcer	49/50	n/d	Cristalloids	n/d	n/d	n/d	Epidural anesthesia	n/d	n/d
Moller 2011	Prospective observational study	Perforated peptic ulcer	117	n/d	Early goal-directed managment	n/d	Active warming with warm air	Goal-directed fluid administration	n/d	n/d	n/d
Nechai 2021	RCT	Cholecystitis	88/101	Preoperative counselling	n/d	Metoclopramide 10 mg at the end of surgery	n/d	n/d	Trocar site infiltration with bupivacaine	n/d	n/d
Pranavi 2022	RCT	Perforation peritonitis	49/61	n/d	n/d	Dexamethasone 4 mg p	n/d	Modified GDFT based on CVP	Epidural anesthesia	n/d	n/d
Pthrikar 2023	RCT	Perforated duodenal ulcer	19/22	n/d	n/d	n/d	n/d	n/d	Epidural analgesia	n/d	n/d
Purushotham 2021	RCT	Trauma laparotomy	30/30	Preoperative counselling	n/d	n/d	Active warming with warm air	n/d	Epidural anesthesia	n/d	n/d
Roulin 2014	retrospective case control study	Colorectal	28/63	preoperative counselling	n/d	Indicated but n/d	Active warming with warm air	n/d	Epidural anesthesia	n/d	n/d
Ruiz-Tovar 2021	Prospective observational	Acute Appendicitis	850	n/d	n/d	Indicated but n/d	Active warming	Goal-directed fluid administration	Trocar site infiltration with ropivacaine	n/d	n/d
Saurabh 2020	RCT	Small bowel surgery	35/35	n/d	n/d	Desamethasone 4 mg	n/d	Goal-directed fluid administration (CVP guided)	Epidural analgesia	n/d	n/d
Shang 2018	Observational case–control study	Obtructive colorectal	318/318	Preoperative counselling	n/d	5-HT3 receptor antagonist + dexamethasone + haloperidol	Active warming with warm air	GDFT	n/d	n/d	n/d
Sharma 2021	RCT	Gastrointestinal surgery	50/50	Preoperative counselling	According to CVP	Indicated but n/d	Active warming	Restricted fluid strict fluid management	Neuroaxial anesthesia	n/d	n/d
Tengberg 2017	Prospective observational study	High-risk abdominal surgery	600/600	n/d	Stroke volume-guided	n/d	n/d	Stroke-volume-guided fluid therapy	TIVA + epidural	n/d	n/d
Wisely 2016	Retrospective case control study	Major abdominal surgery	169/201	n/d	n/d	n/d	n/d	n/d	n/d	n/d	n/d

*n/d* not described

**Table 2:** postoperative protocols description

	Patients	Number of patients	Postoperative						
			Nasogastric tube removal	Liquds per os	oral feeding	Mobilization	iv fluids	Urinary catheter	Drain removal
Burchart 2021	Major abdominal surgery	227	Quick removal	Early	Early feeding	Early	n/d	Early- when removal thoracic epidural	Output < 50 ml
Ceresoli 2023	Gastrointestinal surgery	589	When < 300 ml/d	POD 1	POD 2	Early	When oral intake adequate	When urine output > 0.5 ml/Kg/h	When perforated
Chndan 2021	Perforated peptic ulcer	42/43	When < 300 ml/d	n/d	n/d	POD 0	n/d	When urine output > 1 ml/Kg/h	Output < 100 ml
Gonenc 2013	Perforated peptic ulcer	26/21	Immediately postoperative	POD 1	POD 2	n/d	n/d	Immediately postoperative	n/d
Lohsiriwat 2014	Obtructive colorectal	20/40	POD 2	Early	Early	POD1	n/d	POD3	No routine use
Lohsiriwat 2019	Colorectal	46/14	n/d	n/d	Early feeding	POD1	n/d	n/d	No routine use
Masood 2021	Perforated duodenal ulcer	16/18	POD 0	POD 1	POD 1	POD 0	n/d	POD 0	n/d
Mohsina 2018	Perforated peptic ulcer	49/50	When < 300 ml/d	NPO till bowel sound	One day after first bowel sound	POD 0	n/d	When urine output > 1 ml/Kg/h	when < 100 ml/d
Moller 2011	Perforated peptic ulcer	117	n/d	n/d	n/d	Early	n/d	n/d	n/d
Nechai 2021	Cholecystitis	88/101	End of surgery	2 h after surgery	6 h after surgery	Early	Not indicated	Not indicated	No routine use; only for perforations
Pranavi 2022	Perforation peritonitis	49/61	When < 300 ml/d	NPO till first flatus	One day after first flatus	POD 0	n/d	When urine output > 1 ml/Kg/h	When < 100 ml/d
Pthrikar 2023	Perforated duodenal ulcer	19/22	When < 300 ml/d	POD 1	POD1	POD1	n/d	When urine output adequate	Output < 100 ml
Purushotham 2021	Trauma laparotomy	30/30	POD1	POD 2	POD 1	POD 1	n/d	POD 1	POD 1
Roulin 2014	Colorectal	28/63	Immediately postoperative	POD 0	POD 1	POD1	n/d	POD 1	No
Ruiz-Tovar 2021	Acute Appendicitis	850	n/d	POD 0	POD1	POD0	n/d	n/d	POD1
Saurabh 2020	Small bowel surgery	35/35	When < 300 ml/d	NPO till first flatus	NPO till first flatus	POD0	n/d	When urine output > 1 ml/Kg/h	Output < 100 ml
Shang 2018	Obtructive colorectal	318/318	Immediately postoperative	POD 0	POD 3	POD 1	n/d	Early	No routine use
Sharma 2021	Gastrointestinal surgery	50/50	End of surgery	POD 0	POD 1	POD 0	When oral intake adequate	POD 1	POD 2
Tengberg 2017	High-risk abdominal surgery	600/600	n/d	n/d	n/d	n/d	n/d	n/d	n/d
Wisely 2016	Major abdominal surgery	169/201	n/d	n/d	n/d	n/d	n/d	n/d	n/d

*n/d* not described

Huddart et al. demonstrated that the introduction of a bundle evidence care protocol decreased mortality among patients undergoing emergency laparotomy, with a reduction in delayed diagnosis, increased implementation of goal-directed fluid therapy, and improved restoration of biochemical homeostasis [29]. The bundle protocol consisted in an accurate preoperative assessment with early warning score, early broad-spectrum antibiotics, prompt resuscitation using goal-directed techniques and postoperative ICU admission for all high-risk patients.

Tandberg and colleagues introduced a standardized perioperative care protocol in patients undergoing high-risk emergency abdominal surgery [9]. The study protocol included consultant-led attention and care, early resuscitation and high-dose antibiotics, surgery within 6 h, perioperative stroke volume-guided volume status optimization, standardized analgesic treatment, early mobilization and early oral feeding. Compared with a historical cohort from the same department, the introduction of the protocol lead to a significant reduction in mortality from 21.8 to 15.5%.

An Italian observational multicentric study demonstrated that adherence to the intraoperative protocol items was low. Major determinants of postoperative compliance were minimally invasive surgery and low intraoperative fluid infusions [22].

Several other studies investigated the introduction of enhanced perioperative care protocols in emergency general surgery [10, 12, 14–20, 22, 23, 27]. Each study applied a different protocol in different subsets of patients with contrasting results. Some studies adopted a modified ERAS protocol in patients with obstructive colorectal cancer [13, 14, 16, 19], others on patients with perforated peptic ulcer [10, 15, 17], others on major emergency laparotomy and trauma [12, 18, 20, 22]. Most of the published enhanced recovery programs in emergency surgery focus on the intra- and postoperative phases of the program, reporting no substantial differences in the preoperative care of patients enrolled in ERAS protocols versus standard “not-ERAS” patients. The majority of the existing studies did not report results on adherence to the protocol items; moreover, also data on compliance to the postoperative pathway were lacking.

The results of some of these studies were included in a meta-analysis published by Hajibandeh et al. published in 2020 [30]. Despite the great heterogeneity and the poor quality of the evidence, the results showed a reduction in length of stay, pulmonary complications, postoperative ileus and wound infections. No differences were observed in 30-day rehospitalization and 30-day mortality rates.

Despite the promising results, the implementation of an enhanced perioperative care protocol in emergency general surgery may encounter several obstacles. Patients receiving urgent care typically present to medical teams with a complex situation: their conditions at the time of admission are not optimal and they have extremely heterogeneous characteristics [31, 32]. The acute illness often leads to several physiological derangements secondary to fasting, vomiting, dehydration, augmented capillary permeability, and metabolic imbalance.

Columbus et al. [33] have identified two main critical issues concerning the urgent care field: the diversification of patients and the wide range of possible settings and operative contexts (including the hospital organization and the medical team management). Therefore, efforts should focus on improving the structural and organizational aspects. Dedicated medical personnel training and a widespread standardization of the diagnostic and therapeutic process may improve medical performance and, ultimately, the clinical outcome. A recent study demonstrated that the familiarity between surgeon and anesthetist used to work together improve patients’ outcome [34]. Unfortunately, emergency general surgery is rarely managed by a dedicated staff. Emergency care requires a higher number of specialists and personnel turnover and, therefore, it would lead to wider cross-collaborations and variability in staff composition. In addition, patients undergoing emergency surgery are rarely managed by enhanced recovery-trained anesthetists, surgeons and nurses (working in abdominal surgery), making the development of new treatment protocols very difficult.

The availability of resources is another central tenant to the safe and optimal delivery of surgical care in the emergency setting. For example, laparoscopic facilities or advanced hemodynamic monitoring systems are not universally available, and reported unavailable by some authors, due to logistical issues, timing of surgical interventions (e.g., in office hours vs. after hours), and higher costs [18, 35].

## Methods

The WSES panel promoted the development of this position paper. The work process consisted of two different phases. The first phase was a review of the existing literature about enhanced recovery protocol in emergency general surgery. The second phase was the identification of enhanced recovery protocol items and the development of position statements for each perioperative intervention. This position paper was written according to the WSES methodology [36]. All the statements contained the level of evidence (LoE) available about the topic, graded according to the GRADE methodology. The consensus on the position paper statements was assessed through a web survey (by Google Form) open to all the members of the steering committee and the experts’ panel, as well as the board of governor members of the WSES. The consensus was reached if a statement was associated with ≥ 70% of the agreement. Otherwise, the statement was re-discussed by email or videoconference, modified, and resubmitted to the experts’ vote until consensus was reached. Table 3 summarizes approved statements.

**Table 3:** Position paper statements

	Topic	Statement	Agreement	Level of evidence
Preoperative	Education and counseling	Patient counselling and education should be encouraged and implemented with the aim to explain perioperative risks and post-operative pathway (LoE D)	100%	⨁◯◯◯
	Fluid balance and volemic status	Volemic status should be evaluated and corrected with a goal-directed fluid therapy as soon as possible in the pre-operative phase (LoE B)	100%	⨁⨁⨁◯
	Metabolic balance	Glycemic control should be implemented in all emergency surgery patients in order to prevent both hypo- and hyperglycemia (LoE C)	100%	⨁⨁◯◯
Intraoperative	Postoperative nausea and vomiting (PONV) prevention	PONV prevention with a multimodal approach in emergency setting should be implemented (LoE D)	100%	⨁◯◯◯
	Benzodiazepines	Benzodiazepines should be avoided in emergency anesthetic protocol, in particular in older patients, in order to reduce delirium risk in post-operative period (LoE C)	100%	⨁⨁◯◯
	Opioids	Opioid use should be limited to short-acting drugs in the perioperative period (LoE D)	97.6%	⨁◯◯◯
	Anesthesia depth monitoring	Anesthesia depth monitoring should be implemented in the emergency setting, in order to minimize anesthesia side effects such intra-operative hypotension, increased need for fluids and post-operative delirium (LoE C)	100%	⨁⨁◯◯
	Neuromuscular blockade monitoring	Neuromuscular blockade monitoring should be implemented in order to reduce post-operative morbidity (LoE C)	100%	⨁⨁◯◯
	Multimodal pain control	Multimodal analgesia, with a combination of systemic and loco-regional approach, should be encouraged in emergency setting in order to improve pain control and reduce need for analgesics and opioids (LoE C)	100%	⨁⨁◯◯
	Active warming	Active warming and body temperature monitoring should be encouraged in the emergency setting in order to reduce postoperative morbidity (LoE C)	100%	⨁⨁◯◯
	Fluid Management	Fluids should be managed within a goal-directed fluid therapy strategy with the goal to target the amount of given fluids on patient needs (LoE C)	100%	⨁⨁◯◯
	Minimally invasive surgery	Minimally invasive surgery approach in emergency surgery should be encouraged whenever possible and needed skills are available (LoE C)	100%	⨁⨁◯◯
	Drains	Abdominal drains should be placed for limited indications, including in the presence of gross bacterial contamination and inadequate source control (LoE D)	88.3%	⨁◯◯◯
Postoperative	Analgesia	Multimodal analgesia, using different classes of analgesic and avoiding long-acting opioids, should be recommended in post-operative phase (LoE C)	100%	⨁⨁◯◯
	Early nasogastric tube removal	Nasogastric tube should be removed as soon as possible, even at the end of surgery (LoE D)	97.6%	⨁◯◯◯
	Early mobilization	Early mobilization should be encouraged and stimulated as soon as possible in order to reduce post-operative morbidity (LoE C)	100%	⨁⨁◯◯
	Nutrition and early oral feeding	Early oral feeding should be encouraged and promoted as soon as tolerated by patients (LoE C)	100%	⨁⨁◯◯
	Urinary catheter removal	Urinary catheter should be removed as soon as possible, when urinary output no longer needs to be monitored (LoE C)	100%	⨁⨁◯◯
	Postoperative fluids	Postoperative intravenous. fluids should be minimized and maintained until oral fluid intake is adequate (LoE C)	100%	⨁⨁◯◯
	Antibiotic therapy	Antibiotic therapy should not be continued in case of non complicated intra-abdominal infections, while a short course antibiotic therapy is indicated in case of complicated infection (LoE A)	100%	⨁⨁⨁⨁

### Preoperative interventions

#### Education and counseling

##### Patient counseling and education should be encouraged and implemented to explain perioperative risks and post-operative pathway (LoE D)

Relieving patient anxiety through preoperative counseling is of utmost importance, especially in an emergent situation. Full preoperative counseling, which is known to reduce post-operative stress, pain and anxiety, may not be possible in the emergency setting. Nevertheless, information such as details of the procedure, possible perioperative complications, the need for the creation of a stoma and length of hospitalization should be communicated with patients and their families before the procedure [30, 37]. A recent meta-analysis focusing on the implementation of enhanced recovery protocols in emergency abdominal surgery reported that adapted preoperative counseling was carried out in all of the six included studies. No data about adherence to this counseling were reported [30]. Depending on the urgency of surgery, preoperative education/counseling may not be possible. However, a recently published multidisciplinary experience reported very high compliance (more than 90% 1 year after implementation) with items such as standardized preoperative patient information and bilateral ostomy marking in patients undergoing emergency general surgery [11, 38]. In the case of stoma creation, the implementation of patient education reduced stoma complications and improved postoperative quality of life, reducing the average hospital stay [39]. However, although it seems feasible and of some utility to appropriately counsel patients before emergency procedures, evidence in support of this hypothesis has not been produced yet and the degree of benefit in terms of postoperative recovery has not been measured.

#### Fluid balance and volemic status

##### Volemic status should be evaluated and corrected with goal-directed fluid therapy as soon as possible in the pre-operative phase (LoE B)

The majority of emergency general surgery patients present with fluid derangements, mostly related to acute illness, underlying sepsis, prolonged fasting and vomiting. In this setting, preoperative evaluation of the patient should focus on the volemic assessment to rapidly correct alterations in patients' homeostasis, including stress response, gut dysfunction, insulin resistance, electrolyte imbalances, fluid shifts, SIRS and sepsis with varying degrees of organ dysfunction. Although complete optimization of medical conditions cannot be fully achieved in the emergency setting, adequate intravenous fluid resuscitation in emergency general surgery is crucial and feasible, and it should be attempted in all patients. A prospective randomized trial demonstrated better postoperative outcomes when patients were preoperatively managed with a fixed protocol to reach homeostasis [40]. The adopted protocol defined three targets for the goal-directed crystalloid resuscitation: central venous pressure of 8–12 cmH_2_O, mean arterial pressure > 65 mmHg and urinary output > 0.5 mL/Kg/h. The initial resuscitation should be titrated to the clinical response, such as fluid responsiveness, and not solely guided by a predetermined protocol, with particular attention to the underlying disease. Despite restoring homeostasis should be considered a goal, surgical treatment should not be delayed. According to the indications from the 2016 Surviving Sepsis Guidelines, resuscitation from sepsis-induced hypoperfusion should require at least 30 ml/kg of intravenous crystalloid fluids within the first 3 h [41]. However, in the updated 2021 version of the Guidelines, the strength of this recommendation was downgraded from “strong” to “weak” (quality of evidence: low) and the recommendation was modified in a suggestion [42]. Massive fluid therapy has been challenged in the enhanced recovery approach [9]. Fluid overload should be avoided since it is associated with higher rates of respiratory complications (*i.e.,* pneumonia, pleural effusion, and respiratory failure) and secondary anastomotic leaks. Early, *i.e.,* preoperative, goal-directed fluid therapy in sepsis was the treatment of the experimental arm in a randomized clinical trial enrolling septic patients published in 2001 [43]. Excluding patients who needed immediate surgery from the trial, in-hospital mortality was 30.5% in the group assigned to early goal-directed therapy, as compared with 46.5% in the group assigned to standard therapy (*P* = 0.009). Tendberg et al. developed a perioperative protocol for emergency high-risk abdominal surgery in which stroke volume-guided hemodynamic optimization before surgery was a key element. The study has shown a significant reduction in mortality as well as postoperative length of ICU stay after the introduction of the standardized protocol [9]. Therefore, patients should be carefully evaluated and goal-directed fluid resuscitation should be implemented as soon as possible.

#### Metabolic balance

##### Glycemic control should be implemented in all emergency surgery patients to prevent both hypo- and hyperglycemia (LoE C)

Perioperative hyperglycemia has been demonstrated to be associated with adverse clinical outcomes [44]. The correction of hyperglycemia with insulin administration and the management of glycemia with the implementation of glycaemic control protocols have been shown to reduce hospital complications and decreases mortality in elective general surgery patients [45]. Pre-existing diabetes mellitus, acute illness and physiologic changes accompanying a surgical procedure contribute to the worsening of glycemic control. The resulting hyperglycemia due to an abnormal glucose balance is a risk factor for postoperative complications that include poor wound healing and postoperative infections as well as an increase in morbidity, mortality, intensive care unit admission, and hospital length of stay [46]. Preoperative hyperglycemia has been demonstrated to have a role in postoperative compliance to an enhanced recovery pathway also in emergency surgery patients [22]. However, in emergency general surgery and in critically ill patients the role of hyperglycemia is more debated and less certain. Hyperglycemia could be considered a marker of organ failure and disease severity. A recent network meta-analysis comparing four different target blood glucose concentrations (< 110, 110–144, 144–180, and > 180 mg/dL) in terms of the benefit and risk of insulin therapy found no significant difference in the risk of mortality and infection among four target blood glucose ranges in critically ill patients, but indicated that target blood glucose levels of below 144 mg/dL were associated with a higher risk of hypoglycemia [47]. Although a proactive approach to avoid both hyper- and hypoglycemia should be suggested in emergency patients, close glycemic control is advisable and Institutions should develop their own protocols to treat both hyper and hypoglycemia in critically ill patients.

### Intraoperative interventions

#### Postoperative nausea and vomiting (PONV) prevention

##### PONV prevention with a multimodal approach in an emergency setting should be implemented (LoE D)

Prevention of PONV in elective general surgery has become a key element of enhanced recovery protocols [48]. PONV is very common after general anesthesia and it may negatively impact recovery and short-term outcomes [49]. Several factors are linked to the occurrence of PONV; however, its exact pathophysiology is still unclear [50]. Some risk factors are patient-related such as advanced age, female gender, non-smoking status, pain, and anxiety. Other risk factors are related to the type of operative gastro-intestinal manipulation and vagal stimulation, anesthetics, and opioids [50–52]. Few data are available on emergency patients who frequently complain of nausea and vomiting before surgery in association with anxiety and pain. Several studies investigated the role of different drugs to prevent PONV. The commonest antiemetic drugs are dopamine and serotonin antagonists (e.g., ondansetron) and corticosteroids (e.g., dexamethasone) [53–56]. Pre-emptive anesthesia was associated with better pain control and reduction in PONV [57, 58].

Other suggested interventions are opioid-sparing anesthesia and avoidance of volatile anesthetics. Unfortunately, the vast majority of evidence is based on elective surgery and very few data are available on emergency general surgery. The emergency setting is associated with more fear, anxiety, pain and, probably, nausea even before surgery. Nevertheless, prevention of PONV should be implemented also in emergency general surgery. Among the interventions suggested, there are opioid-sparing anesthesia, avoidance of volatile anesthetics and a multimodal approach to pharmacological prevention.

#### Anesthesia and analgesia

General anesthesia warrants proper analgesia, amnesia and muscle relaxation. The ideal general anesthesia protocol should target all these goals, but it should also reduce the need for intraoperative fluids, reduce post-operative residual effects, such as PONV and delirium and it should permit rapid awakening. Several interventions have been implemented to optimize the intraoperative management of the patient. Whether anesthesia should be maintained by a totally intravenous approach or with inhalation drugs still remains uncertain and no recommendations can be made [59]

#### Benzodiazepines

##### Benzodiazepines should be avoided in the emergency anesthetic protocol, in particular in older patients, to reduce delirium risk in the postoperative period (LoE C)

The incidence of delirium in the postoperative period has an important impact on clinical outcomes including higher mortality, functional decline, prolonged hospitalizations and risk for institutionalization [60]. Upon the several risk factors for development, that include acute illness and pain management, medications adopted also for general anesthesia play an important role [61]. For these reasons, anesthetic protocols should focus on reducing the use of these medications. Benzodiazepines have been linked with the development of delirium in the postoperative period, with a marked effect in elderly and frail patients [62, 63]. Despite the potential beneficial effects in treating preoperative anxiety, these drugs should be avoided.

#### Opioids

##### Opioid use should be limited to short-acting drugs in the perioperative period (LoE D)

Opioids are related to several adverse effects such as nausea, vomiting, respiratory depression, sedation and postoperative ileus. Despite their important role in pain management, the undesired effects may impact negatively on patients' recovery. Some experiences exist about opioid-free anesthesia, with the claim of more patient safety [64]. For this reason, opioids use should be limited to short-acting drugs avoiding morphine to minimize residual effects and to warrant rapid recovery [65].

#### Anesthesia depth monitoring

##### Anesthesia depth monitoring should be implemented in the emergency setting, to minimize anesthesia side effects such intra-operative hypotension, increased need for fluids and postoperative delirium (LoE C)

To reduce all the detrimental effects of general anesthetics, such as cognitive effects and vasoactive depressing activity, titrating the minimal needed drug dose guided by the depth of anesthesia monitoring has been recommended. Monitoring of anesthesia depth could be guided by the bispectral index (BIS) or other techniques based on electrical brain activity (EEG). Anesthesia depth monitoring has been demonstrated to be associated with a lower incidence of postoperative delirium and with decreased morbidity [66–68]. Moreover, depth monitoring has been demonstrated to be associated also with a higher intraoperative mean arterial pressure, possibly reducing the need for fluid administration to maintain adequate systemic perfusion [69].

#### Neuromuscular blockade monitoring

##### Neuromuscular blockade monitoring should be implemented to reduce post-operative morbidity (LoE C)

Neuromuscular blockade is needed during abdominal surgery to improve surgical exposure. A post-operative residual neuromuscular block is a risk factor for morbidity and mortality, conditioning weakness of airway muscles, airway obstruction and aspiration with consequent increased postoperative pulmonary complications [70]. Residual neuromuscular block has been reported in up to 40% of patients treated with neuromuscular blocking agents [71]. Adopting strategies such as the qualitative monitoring of the peripheral muscular blockade as the train of four (TOF) has been demonstrated to significantly reduce the residual blockade at the end of anesthesia [72]. Monitoring of the neuromuscular blockade is therefore recommended to avoid potential side effects.

#### Multimodal pain control

##### Multimodal analgesia, with a combination of systemic and loco-regional approaches, should be encouraged in the emergency setting to improve pain control and reduce the need for analgesics and opioids (LoE C)

Pain is one of the limitations to patient recovery after surgery. Standard general anesthesia warrants analgesia during surgery, but has no effect on pain control after surgery, requiring drug administration with possible detrimental effects such as opioids. Multimodal analgesia has been proposed to manage pain with several different treatments reducing the need for systemic opioids and avoiding their potential side effects [73]. The association of general and locoregional analgesia has been demonstrated also to reduce the incidence of postoperative delirium [74].

Thoracic epidural analgesia (TEA) has been demonstrated to be superior to systemic opioids in pain management in open elective abdominal surgery [75]. A recent Scandinavian population study reported that epidural analgesia was adopted in emergency general surgery in less than one third of patients; epidural analgesia was associated with lower 90-day mortality probably due to a reduction in paralytic ileus and pain that most likely allowed an early mobilization and coughing [76]. TEA was included in an emergency general surgery enhanced recovery protocol that demonstrated a significant reduction in mortality, despite the adherence to this specific item was not reported [9]. Spinal analgesia has been proposed as an alternative to epidural analgesia in patients treated with minimally invasive colorectal surgery: the administration of long-acting local anesthetics and opioids warrant pain control in the first postoperative hours allowing early mobilization. Moreover, it has been associated with a lower risk of hypotension and fluid overload [77]. However, spinal and epidural anesthesia should be considered with caution in septic patients.

Surgical incision is one of the main responsible of postoperative pain. To manage this pain, abdominal wall blockade such as the Transversus abdominis plane (TAP) block has been proposed. The adjunct of abdominal wall blocks to general anesthesia has been demonstrated to have beneficial effects on pain control during the first 24 h and to allow faster recovery and better hemodynamic control in elective abdominal surgery [78–81]. Of note, the TAP block can be performed both ultrasound-guided and laparoscopy-guided [82]. Currently, no studies focus on the performance of the TAP block in emergency general surgery. However, abdominal wall blocks should be considered in a multimodal analgesic approach.

#### Active warming

##### Active warming and body temperature monitoring should be encouraged in the emergency setting to reduce postoperative morbidity (LoE C)

Body temperature plays an important role in several pathophysiologic mechanisms Hypothermia typically occurs during general and locoregional anesthesia due to vasodilatation and a rapid redistribution of heat from the core to peripheral districts. Moreover, several anesthetic drugs impair thermoregulatory control, further contributing to the maintenance of hypothermia. Finally, the development of hypothermia is facilitated by direct heat loss deriving from the surgical exposure of the abdominal cavity and by the low operating theater temperature. Importantly, perioperative hypothermia implicates an increased risk of surgical site infection, morbidity and mortality. Moreover, hypothermia may alter drug metabolism and it is also associated with an increased risk for coagulopathy and a consequent increased blood loss [83]. Body temperature monitoring is therefore mandatory and allows temperature correction with active warming. Active warming, ideally starting before the entrance to the operating room, has been recognized as one of the core items of the enhanced recovery pathway and its implementation significantly reduced postoperative morbidity [84, 85].

#### Fluid management

##### Fluids should be managed within a goal-directed fluid therapy strategy to target the amount of given fluids on patient needs (LoE C)

General anesthetics lead to dose-depend myocardial depression and systemic vasodilatation. The associated increased venous capacitance leads to a relative hypovolemia that, along with myocardial depression, might lead to hypotension, and organ hypoperfusion with the related consequences. Therefore, during surgery, fluids are frequently administered to maintain an adequate intravascular volume status and systemic perfusion. However, both hypovolemia and hypervolemia are associated with postoperative morbidity and several studies demonstrated the J-shaped relation between intraoperative fluids administered and postoperative morbidity [86–88]. Intraoperative fluid management should therefore be balanced, giving the needed amounts of fluids to warrant euvolemia and systemic perfusion, but avoiding fluid overload [89, 90]. Fluid overload is associated with several detrimental effects related to tissue edema. Increased interstitial fluids might impair gas exchange with consequent respiratory failure and foster the development of pneumonia. Moreover, fluid overload is associated with bowel edema and postoperative ileus, conditioning a delayed recovery of GI function [91]. For these reasons in elective surgery, a restrictive fluid strategy has been proposed, with the target of a near-zero fluid balance during surgery and a limited amount of fluids given (generally around 3 mL/Kg/h) [92]. This approach is valid under the condition that patients arrive at the surgery in perfect homeostasis without fluid derangements.

Several factors may worsen and make fluid management in emergency surgery patients more difficult. Increased vascular permeability related to acute illness, preoperative fasting, preoperative dehydration and blood loss may dramatically increase the need for intraoperative fluids compared to elective surgical patients. In this complex scenario, goal-directed fluid therapy has been proposed to titrate and balance the amount of fluids. Fluid therapy should be guided by hemodynamic monitoring systems, ideally capable of monitoring dynamic parameters, such as cardiac output, stroke volume variation, pulse pressure variation and stroke volume variation [93, 94]. The implementation of an intraoperative goal-directed fluid strategy, associated with restrictive fluid regimens and the early adoption of vasopressors to maintain adequate circulating volumes has been demonstrated to significantly reduce perioperative morbidity [94]. While only few studies exist on fluid management during general emergency surgery, available evidence derived from elective surgery and current pathophysiological understanding strongly underlines the importance of reasoned fluid management during emergency surgery. In the emergency setting, a recent study highlighted the importance of fluid therapy, with a negative correlation between increasing intraoperative fluids given and patients' recovery [22].

#### Minimally invasive surgery

##### Minimally invasive surgery approach in emergency surgery should be encouraged whenever possible and needed skills are available (LoE C)

Reducing surgical stress is the cornerstone of an enhanced perioperative care protocol. The use of minimally invasive surgery in elective major surgery has been demonstrated to reduce inflammation, improve pulmonary function, and facilitate GI function with a consequent reduction in morbidity and length of stay [95–97]. Minimally invasive surgery, even within an enhanced recovery pathway, has been associated with a faster recovery when compared with open surgery [98]. In emergency major abdominal surgery, such as repair for perforated peptic ulcer and colorectal surgery invasive minimally techniques have been associated with better clinical outcomes with a lower mortality and length of stay [99–101]. A population study on the commonest abdominal surgical emergencies in the USA demonstrated an increasing trend of a laparoscopic approach. Minimally invasive surgery was associated with lower mortality, surgical site infection rate and length of stay. However, minimally invasive surgery in major interventions such as peptic ulcer repair and small bowel obstruction was adopted in less than 40% and 10%, respectively [102]. Data from the national emergency laparotomy audit (NELA) from the U.K. demonstrated that laparoscopy is adopted in less than 20% of major surgeries [99]. Existing data demonstrated the beneficial effect of minimally invasive surgery but also its poor diffusion among surgeons with several difficulties [103]. A recent prospective study identified minimally invasive surgery as the major determinant of postoperative compliance to an enhanced recovery protocol [22]. Efforts should be made to implement laparoscopy in emergency general surgery daily practice.

#### Drains

##### Abdominal drains should be placed for limited indications, including in the presence of gross bacterial contamination and inadequate source control (LoE D)

The routine positioning of a peritoneal drain after elective major colorectal surgery has been demonstrated to be ineffective in preventing surgical complications and is not recommended [104]. Moreover, the presence of a drain has been identified as one of the main failure predictors of an enhanced recovery pathway, both in elective and emergency surgery [22, 105]. Drain in emergency general surgery is justified by a clear rationale, in case of contaminated surgical field and intra-abdominal infections. Few experiences exist about avoiding drains in emergency general surgery: some studies focused on the introduction of enhanced perioperative care protocol on colorectal emergencies (obstructions) demonstrated better results avoiding the drain (along with other interventions) [13, 14, 16]; other studies demonstrated the safety of an early removal in perforated peptic ulcer and trauma [15, 20]. Evidence quality is very low to recommend avoiding abdominal drains, but we believe drains should be placed only in case of gross abdominal contamination and high risk for collection and abdominal abscess.

### Postoperative care

#### Analgesia

##### Multimodal analgesia, using different classes of analgesics and avoiding long-acting opioids, should be recommended in the postoperative phase (LoE C)

Proper analgesia and pain control are key elements of a patient’s recovery after surgery. The control of pain in the postoperative period is the result of many several factors related to patients' characteristics, invasiveness of surgical intervention, the underlying diagnosis and adopted intra- and post-operative analgesia techniques. Perioperative management should be focused on maximizing the effect of pain control and avoiding the side effects of drugs. The use of long-acting opioids, such as morphine, should be ideally avoided also in the postoperative period. Indeed, avoiding opioids has been demonstrated to facilitate mobilization and to fasten GI function recovery [65]. The treatment of pain should be multimodal and tailored to patients’ conditions, according to available skills [106].

#### Early nasogastric tube removal

##### The nasogastric tube should be removed as soon as possible, even at the end of surgery (LoE D)

According to a reactive policy, the nasogastric tube (NGT) was traditionally removed after GI function recovery to prevent PONV and inhalation. Enhanced recovery protocols recommend the removal of NGT at the end of elective surgery. This practice reduced pulmonary complications and promoted GI function recovery [107]. Preliminary studies carried out in patients with obstructive colorectal cancer or perforated peptic ulcer reported a high patient compliance to NGT removal at the end of surgery [17, 19, 108]. Other studies suggested removing the NGT when the output was less than 300 ml [15, 18]. When patients are managed according to enhanced recovery protocols, the early removal of NGT is safe and should be implemented in clinical practice.

#### Early mobilization

##### Early mobilization should be encouraged and stimulated as soon as possible to reduce post-operative morbidity (LoE C)

Prolonged immobilization is associated with insulin resistance, thromboembolic events and respiratory complications [109]. Several studies reported that early mobilization after surgery reduced overall morbidity and shortened the length of hospital stay [110–113]. Several factors can negatively impact on patient’s mobilization such as abdominal drain, urinary catheter, suboptimal pain control, prolonged i.v fluids, and patient's motivation. In emergency surgery, different protocols have been proposed targeting mobilization the same day of surgery [15, 18] or on postoperative day 1 [14, 16, 19]. According to existing evidence, patient mobilization should be encouraged as early as possible, along with all the interventions that could facilitate it, such as proper pain control, and the early removal of urinary catheter and drains.

#### Nutrition and early oral feeding

##### Early oral feeding should be encouraged and promoted as soon as tolerated by patients (LoE C)

The close relationship between preoperative nutritional status and surgical outcomes has been extensively reported in elective surgery, where tailored nutritional and prehabilitation programs can be planned before the operation [114]. Postoperative fasting has been demonstrated to be harmful in elective surgery with delayed recovery and increased complications[2, 115, 116]. Oral feeding can be resumed early after surgery regardless of bowel canalization, whether removal of the nasogastric tube, PONV prophylaxis, near zero fluid balance, early mobilization, and pain control have been carried out according to enhanced recovery protocol. Patients undergoing emergency surgery often have an altered metabolic status, with dehydration and several derangements such as prolonged fasting, vomiting, impairment of GI function, and fluid loss related to the acute illness. The great condition's heterogeneity in emergency surgery patients makes quite impossible to standardize the timing of oral feeding recovery. However, studies carried out in patients with perforated peptic ulcer or obstructive colorectal cancer demonstrated both feasibility and safety of early oral feeding [19, 20, 117]. Perioperative nutritional intervention should be therefore tailored to the patient's conditions adopting as the target the earliest possible recovery.

#### Urinary catheter removal

##### Urinary catheter should be removed as soon as possible when urinary output no longer needs to be monitored (LoE C)

Urinary output monitoring is a key element to assess patients' volemic status and to guide goal-directed fluid therapy. In emergency surgery patients, the urine output target should be 0.5 ml/Kg/h. Different policies about the timing of catheter removal have been proposed: immediately after surgery in a randomized study on perforated peptic ulcer patients [17], on postoperative day 1 [19, 20] or according to urinary output (> 1 ml/Kg/h) [15, 18]. Regardless, the urinary catheter should be removed as early as possible after reaching the minimum urinary output target to facilitate mobilization and reduce infections.

#### Postoperative fluids

##### Postoperative intravenous fluids should be minimized and maintained until oral fluid intake is adequate (LoE C)

Fluid therapy should be targeted to restore the euvolemic status and to maintain adequate hydration and tissue perfusion until the oral intake can be restarted. As reported for operative management, fluid therapy can be harmful if too many or too few fluids are given [86–88]. Following elective colorectal surgery i.v. fluids should be stopped on postoperative day one. Studies performed on emergency surgery patients did not report on timing to stop i.v fluids; however, infusions should be tailored to patient conditions, giving the minimum fluid amount to restore and maintain euvolemia and to obtain adequate perfusion.

#### Antibiotic therapy

*Antibiotic therapy should not be continued in case of non-complicated intra-abdominal infections, while a short course antibiotic therapy is indicated in case of complicated infection (LoE A).*

A large part of emergency patients undergo surgery for intra-abdominal infections; therefore, antibiotic therapy is a cornerstone of treatment along with surgical source control**.** The need for antibiotics during the postoperative period may contribute to delaying patient recovery, as an obstacle to active mobilization and i.v. infusions suspension; moreover, prolonged antibiotic therapies may have a role in delaying home return. Postoperative antibiotic therapy should be reserved for patients with complicated intra-abdominal infections. In these patients, a short therapy (3–5 days) after adequate surgical source control is not inferior when compared to longer therapy [118–120]. In non-complicated infections, antibiotic therapy should be stopped at the end of surgery if the source control is adequate.

The majority of patients presenting with a severe infection who initially require IV therapy can be switched to oral therapy after 24–48 h provided that they are improving clinically and can tolerate an oral formulation. The switch from IV to oral route should be encouraged.

### Research agenda

The present position paper highlights the great heterogeneity of protocols adopted and the lack of good-quality evidence supporting the implementation of enhanced recovery pathway in emergency general surgery. Further studies on this topic should address:

- The definition of the safety, feasibility and effectiveness of each perioperative intervention.
- The definition of a standardized enhanced recovery protocol for emergency general surgery procedures
- The selection of patients who may benefit from an enhanced recovery pathway and the clinical scenarios in which enhanced recovery pathway could be applied.

## Conclusions

Enhanced perioperative care, similar to elective surgery, should be implemented in emergency general surgery. One of the key elements for the success of the enhanced pathways is the multimodal approach involving surgeons, anesthetists, ICU physicians, nurses, patients and patient families. Available evidence suggests future required research on the implementation of enhanced recovery pathways in clinical practice.

## Data Availability

Data are available under request to the corresponding author.
